# Risk of SARS-CoV-2 Acquisition in Health Care Workers According to Cumulative Patient Exposure and Preferred Mask Type

**DOI:** 10.1001/jamanetworkopen.2022.26816

**Published:** 2022-08-15

**Authors:** Tamara Dörr, Sabine Haller, Maja F. Müller, Andrée Friedl, Danielle Vuichard, Christian R. Kahlert, Philipp Kohler

**Affiliations:** 1 Division of Infectious Diseases and Hospital Epidemiology, Cantonal Hospital St Gallen, St Gallen, Switzerland; 2 Hirslanden Clinic, Zurich, Switzerland; 3 Division of Infectious Diseases and Hospital Epidemiology, Cantonal Hospital Baden, Baden, Switzerland; 4 Division of Infectious Diseases and Hospital Epidemiology, Thurgau Hospital Group, Muensterlingen, Switzerland; 5 Swiss National Centre for Infection Prevention (Swissnoso), Berne, Switzerland; 6 Department of Infectious Diseases and Hospital Epidemiology, Children’s Hospital of Eastern Switzerland, St Gallen, Switzerland

## Abstract

This cohort study compares the risk of infection with SARS-CoV-2 among health care workers by mask preference and level of patient exposure.

## Introduction

Health care workers (HCWs) are at increased risk for acquiring SARS-CoV-2 infection,^1^ raising the issue of adequate protective measures. Although scientific evidence regarding the benefit of respirator vs surgical masks is sparse,^2,3^ a previous study has suggested that respirator masks (ie, FFP2) may offer additional protection to HCW with frequent COVID-19-patient exposure.^4^ In this follow-up study, we analyzed the SARS-CoV-2 risk for HCWs depending on cumulative exposure to patients with COVID-19 and assessed whether this risk can be modulated by the use of respirator compared with surgical masks.

## Methods

This cohort study was approved by the ethics committee of Eastern Switzerland. Written informed consent was obtained from participants. The study included volunteer HCWs from 7 health care networks in Northern and Eastern Switzerland.^5^ Baseline data (collected in September 2020) included anthropometric characteristics and job descriptions. In weekly follow-up evaluations during 12 months, participants indicated results of symptom-based SARS-CoV-2 nasopharyngeal swabs, exposures, and risk behavior (eMethods and eTable in the Supplement). In September 2021, participants indicated which mask type they had used in contact (if any) with patients with COVID-19 in the last 12 months outside of aerosol-generating procedures (among surgical mask only, both mask types, and respirators only). To assess cumulative patient exposure, we multiplied self-reported number of contacts with patients with COVID-19 (range, 0-100) and mean contact duration (range, 1-60 minutes). Cumulative patient exposure was grouped into 8 categories defined by powers of 2. At baseline, in January and September 2021, participants were screened for antinucleocapsid antibodies.^5^

The main outcome was SARS-CoV-2 infection during follow-up, ie, self-reported positive nasopharyngeal swab and/or antinucleocapsid seroconversion from baseline. Odds ratios (ORs) for the increase in SARS-CoV-2 positivity per doubling of contact time were calculated separately for HCWs using respirator masks only and those who used only surgical or both mask types. We used logistic regression to adjust for a priori–defined covariables and included networks as random effects (eMethods and eTable in the Supplement). Sensitivity analysis was performed excluding participants with positive households. R, version 3.6.1 (R Foundation for Statistical Analysis) was used for statistical analysis; 2-sided, unpaired *P* values <.05 were considered significant. This report follows STROBE reporting guideline for observational studies.

## Results

We included 2919 HCWs (median age, 43 years (range, 18-73 years); 749 participants (26%) were infected with SARS-CoV-2. SARS-CoV-2 positivity was 13% in HCWs without patient exposure. For those exposed to patients, positivity was 21% for HCWs using respirator masks and 35% for those using surgical/mixed masks (OR, 0.49; 95% CI, 0.39-0.61), showing an increase for surgical/mixed mask users (OR, 1.21; 95% CI, 1.15-1.28) and respirator mask users (OR, 1.15; 95% CI, 1.05-1.27) across categories of patient exposure (Figure). Variables associated with SARS-CoV-2 infection in multivariable analysis included a positive household contact (OR, 7.79; 95% CI, 5.98-10.15), exposure to patients (OR, 1.20 per category of cumulative contact; 95% CI, 1.14-1.26), respirator use (OR, 0.56; 95% CI, 0.43-0.74), and SARS-CoV-2 vaccination (OR, 0.55; 95% CI, 0.41-0.74) (Table). Similar results were obtained in sensitivity analysis.

**Figure.**
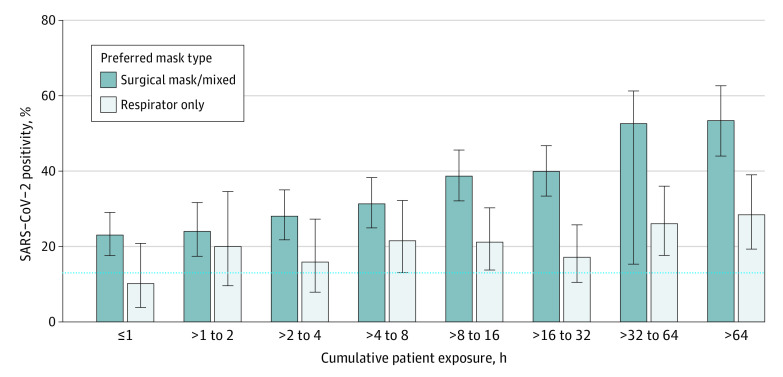
SARS-CoV-2 Positivity in Health Care Workers Depending on Cumulative Patient Exposure and Mask Type Incidence of SARS-CoV-2 in health care workers depending on cumulative patient exposure during 12 months. Dashed line indicates SARS-CoV-2 positivity in participants without patient contact. Error bars indicate 95% CIs.

**Table.** Participant Characteristics by SARS-CoV-2 Status and Results of Logistic Regression Analyses Regarding SARS-CoV-2 Risk

Variable	SARS-CoV-2 status, No. (%)		Univariable analysis^a^		Multivariable analysis^a^	
	Negative (n = 2170)	Positive (n = 749)	OR (95% CI)	*P* value	OR (95% CI)	*P* value
Baseline						
Age, median (range), y	43.2 (18-73)	40.6 (18-66)	0.98 (0.97-0.99)	<.001	0.99 (0.98-1.01)	.35
BMI, median (range)	24.4 (14.3-65.8)	24.3 (15.8-44.6)	1.00 (0.98-1.01)	.62	1.00 (0.98-1.03)	.75
Sex						
Female	1701 (78.4)	597 (79.7)	0.96 (0.78-1.18)	.69	0.76 (0.57-1.00)	.05
Male	469 (21.6)	152 (20.3)	[Reference]			
Pregnancy	46 (2.1)	25 (3.3)	1.52 (0.92-2.51)	.10	0.64 (0.34-1.20)	.16
Active smoker (vs never/former)	323 (14.9)	77 (10.3)	0.68 (0.52-0.88)	.004	0.68 (0.49-0.95)	.02
At least 1 comorbidity	898 (41.4)	298 (39.8)	0.96 (0.81-1.14)	.62	1.02 (0.82-1.27)	.85
Work-related factors						
Cumulative patient contact (OR per category), h						
0	720 (33.2)	110 (14.7)	1.22 (1.18-1.26)	<.001	1.20 (1.14-1.26)	<.001
>0-1	230 (10.6)	59 (7.9)				
>1-2	150 (6.9)	45 (6.0)				
>2-4	189 (8.7)	63 (8.4)				
>4-8	198 (9.1)	79 (10.5)				
>8-16	212 (9.8)	104 (13.9)				
>16-32	218 (10.0)	105 (14.0)				
>32-64	135 (6.2)	96 (12.8)				
>64	118 (5.4)	88 (11.7)				
Always respirator (vs surgical/mixed mask use)^b^	506 (23.3)	132 (17.6)	0.57 (0.45-0.73)	<.001	0.56 (0.43-0.74)	<.001
Working ≥80% FTE	1130 (52.1)	430 (57.4)	1.30 (1.07-1.50)	.007	1.39 (1.10-1.77)	.006
Working in intensive care	189 (8.7)	67 (8.9)	1.05 (0.78-1.41)	.75	0.82 (0.57-1.16)	.26
Hospital canteen visit once weekly or more (vs less)	1418 (65.3)	490 (65.4)	1.01 (0.85-1.21)	.88	1.15 (0.91-1.45)	.23
Nonwork-related factors						
SARS-CoV-2 vaccination	1915 (88.2)	577 (77.0)	0.49 (0.39-0.60)	<.001	0.55 (0.41-0.74)	<.001
Positive household contact	165 (7.6)	314 (41.9)	8.82 (7.09-11.0)	<.001	7.79 (5.98-10.15)	<.001
Always wearing a mask outside work	162 (7.5)	69 (9.2)	1.25 (0.93-1.68)	.15	1.33 (0.91-1.93)	.14

Abbreviations: BMI, body mass index (calculated as weight in kilograms divided by height in meters squared); FTE, full-time equivalent; OR, odds ratio.

^a^ Generalized mixed-effects model (with logit link) using health care network as random effect.

^b^ In contact with patients with COVID-19 outside of aerosol-generating procedures.

## Discussion

In this study, SARS-CoV-2 positivity in HCWs was associated with cumulative COVID-19 patient exposure. The odds of being SARS-CoV-2–positive were reduced by more than 40% in individuals using respirators irrespective of cumulative exposure, even after adjusting for multiple work- and nonwork-related covariables.

These data suggest a dose-response association between COVID-19-patient exposure and risk of SARS-CoV-2 infection in HCWs. The presumable protection conferred by respirator use is in line with previous data.^1,4^ Self-reporting of preferred mask type and residual confounding are potential study limitations.

Consequent use of respirators and SARS-CoV-2 vaccination might substantially decrease the work-related risk for HCWs exposed to patients with COVID-19. Whether these results are applicable to newer viral variants, which are more contagious and less neutralized by most vaccines,^6^ remains to be seen.

## Notes

Supplement. **eMethods.** Detailed Methods **eTable.** A Priori–Defined Covariables, Including Definitions, Answer Levels, and Source Questionnaire
eReferences
