# Supplementary material for: Risk of SARS-CoV-2 Acquisition in Health Care Workers According to Cumulative Patient Exposure and Preferred Mask Type
Source: JAMA Netw Open. 2022 Aug 15;5(8):e2226816. doi: 10.1001/jamanetworkopen.2022.26816 (PMC9379739; doi:10.1001/jamanetworkopen.2022.26816)
Supplement: Supplement. — eMethods. Detailed Methods eTable. A Priori–Defined Covariables, Including Definitions, Answer Levels, and Source Questionnaire eReferences [file jamanetwopen-e2226816-s001.pdf]

Supplementary Online Content

Dörr T, Haller S, Müller MF, et al. Risk of SARS-CoV-2 acquisition in health care workers according to cumulative patient exposure and preferred mask type. *JAMA Netw Open*. 2022;5(8):e2226816. doi:10.1001/jamanetworkopen.2022.26816

eMethods. Detailed Methods

eTable. A Priori–Defined Covariables, Including Definitions, Answer Levels, and Source Questionnaire

eReferences

This supplementary material has been provided by the authors to give readers additional information about their work.

© 2022 Dörr T et al. *JAMA Network Open.*

eMethods. Detailed Methods

Study participants

All healthcare workers (HCW) aged 18 or older, irrespective of patient contact, were approached for study participation. No exclusion criteria were applied.

Validation of nasopharyngeal swab results

Detection of SARS-CoV-2 from nasopharyngeal swabs (NPS) was made by PCR or rapid antigen test, depending on the method used in the participating institutions. To verify the completeness and accuracy of self-reported NPS results, all self-reported positive tests and a random sample of negative test results were cross-checked with the database of the division of occupational health for a subgroup of HCWs from the largest participating institution.
We found that 150 out of 174 presumable positive NPS were indeed documented in the database of the division of occupational medicine. The remaining HCWs most likely had a positive NPS outside of their working place. On the other hand, none of the randomly selected 175 HCWs reporting only negative NPS results was found to have a positive NPS in the database[1].

Univariable and multivariable analysis

Co-variables were a priori selected based on their association with the SARS-CoV-2 risk in HCW found in previous studies[2] (**Table S1**). We used a generalized mixed effects model with logit link to assess the univariable and multivariable association of these variables with the outcome SARS-CoV-2 positivity. Healthcare networks were treated as random effects. R statistical software Version 3.6.1 was used for statistical analysis; p-values of <.05 were considered significant.

© 2022 Dörr T et al. *JAMA Network Open.*

eTable. A Priori–Defined Covariables, Including Definitions, Answer Levels, and Source Questionnaire

Variable name (unit)	Definition	Levels	Questionnaire
**Anthropometrics/baseline health**			
Age (years)	Age at time of last serology	Number	Follow-up
Body mass index (kg/m2)	BMI at time of study entry	Number	Baseline
Sex	According to participants choice	Female; Male (Ref)	Baseline
Pregnancy	Pregnant at time of study entry	Yes; No (Ref)	Baseline
Smoking status	Smoking status at time of study entry	Active; Never/former (Ref)	Baseline
Comorbidities	Presence of any of the following: arterial hypertension, diabetes, cancer, pulmonary disease, rheumatologic disease, other	Yes; No (Ref)	Baseline
**Work-related factors**			
Fulltime work ≥80%	Fulltime equivalent ≥80% at study entry	Yes; No (Ref)	Baseline
Works in intensive care	Works in intensive care at study entry	Yes; No (Ref)	Baseline
Cumulative patient exposure (in hours, h)	(Number of COVID-19 patients which participant was exposed to since beginning of the study) x (average duration of patient contact)	Categorized by power of two (i.e. 1h, >1-2h; >2-4h; >4-8h; >8-16h; >16-32h; >32-64h; >64h); no patient contact (Ref)	Follow-up
Mask type	Mask type preferentially used during COVID-19 patient contact during study period (outside of aerosol-generating procedures)	Always respirator; mixed/mostly surgical masks (Ref)	Follow-up
Hospital cafeteria	Use of hospital cafeteria	At least weekly; less than weekly (Ref)	Follow-up
**Non-work related exposures and behaviours**			
SARS-CoV-2 vaccination	Having had ≥1 SARS-CoV-2 vaccination	Yes; No (Ref)	Follow-up
Positive household contact	At least one household contact with positive SARS-CoV-2 swab at any time point	Yes; No (Ref)	Follow-up
Always wearing a mask outside of work	Wearing mostly a surgical mask in public locations	Yes; No (Ref)	Follow-up

Kg, Kilogram; m, Meter; Ref, Reference

© 2022 Dörr T et al. *JAMA Network Open.*

eReferences

1. Kohler P, Güsewell S, Seneghini M, et al. Impact of baseline SARS-CoV-2 antibody status on syndromic surveillance and the risk of subsequent COVID-19—a prospective multicenter cohort study. *BMC Med*. 2021;19(1):270. doi:10.1186/s12916-021-02144-9

2. Kahlert CR, Persi R, Güsewell S, et al. Non-occupational and occupational factors associated with specific SARS-CoV-2 antibodies among hospital workers – A multicentre cross-sectional study. *Clin Microbiol Infect*. Published online May 2021:S1198743X21002366. doi:10.1016/j.cmi.2021.05.014

© 2022 Dörr T et al. *JAMA Network Open.*
